# Revisiting development strategy under climate uncertainty: case study of Malawi

**DOI:** 10.1007/s10584-024-03733-2

**Published:** 2024-05-31

**Authors:** Askar Mukashov, Timothy Thomas, James Thurlow

**Affiliations:** International Food Policy Research Institute, 1201 Eye St NW, Washington, DC 20005 USA

**Keywords:** Climate uncertainty, Development strategy, CGE modeling, D58, C68, Q54, O20

## Abstract

This paper analyzes the effectiveness of agriculture-led versus non-agriculture-led development strategies under climate-induced economic uncertainty. Utilizing Malawi as a case study, we introduce the application of Stochastic Dominance (SD) analysis, a tool from decision analysis theory, and compare the two strategies in the context of weather/climate-associated economic uncertainty. Our findings suggest that an agriculture-led development strategy consistently surpasses its non-agriculture-led antagonist in poverty and undernourishment outcomes across almost all possible weather/climate scenarios. This underscores that, despite increasing exposure of the entire economy to weather/climate uncertainty, agriculture-led development remains the optimal strategy for Malawi to reduce poverty and undernourishment. The study also endorses the broader use of SD analysis in policy planning studies, promoting its potential to integrate risk and uncertainty into policymaking.

## Introduction

Studies on economic development typically conclude that agricultural growth plays a key role in countries’ early development due to its strong linkages to poverty reduction (see, e.g. Diao et al. 2010; Valdés and Foster 2010; Christiaensen et al. 2011; Klasen and Reimers 2017). Accordingly, most low-income countries prioritize agriculture in their national development strategies and investment plans (see, e.g., Kamenya et al. 2018; Makombe et al. 2019; Chitiga et al. 2020). However, agriculture is also particularly sensitive to climate variability, which is expected to increase under climate change (Omotoso et al. 2023). Incorporating climate uncertainty into the development discourse is thus a research and policy priority.

Numerous studies have analyzed the potential consequences of various climate change scenarios on economic development in African countries, with more recent works acknowledging the uncertain nature of weather and climate projections and quantifying the associated economic uncertainty. For instance, Arndt and Thurlow (2014), Arndt et al. (2014), and Siddig et al. (2020) provide valuable insights into the economic impacts of climate uncertainty in Mozambique, Malawi, and Sudan, respectively. Despite this progress, there has been insufficient consideration of a comparison of alternative development scenarios in the context of climate uncertainty.

One reason why previous studies have not yet jointly analyzed alternative development strategies under climate uncertainty is difficulties in representing and modeling the full range of possible climate and weather realizations. Another reason is the challenge of comparing the different growth strategies when their outcomes are uncertain, as the ranking of development strategies depends on the underlying weather/climate assumptions.

In this paper, we propose the use of Stochastic Dominance (SD) analysis from decision theory to compare development scenarios under climate uncertainty. SD analysis aims to rank competing options by comparing their Cumulative Distribution Functions (CDFs) that characterize their uncertainty (Levy 1992). Although commonly used in finance (Levy 1992) and occasionally in some micro-level experimental studies,^1^ the application of CDF and SD analysis in policy planning models remains rare, often relegated to the robustness checks in technical appendices. An illustrative case is the study by Islam and Braden (2006), which developed a floodplain management policy model for Bangladesh and conducted a robustness test of the base (natural) scenario’s performance over other floodplain management scenarios in the appendix of the article. In this context, our paper distinctively contributes to the methodology by focusing on CDF and SD analysis methods as a means of comparing the uncertainty profiles of alternative development options.

We demonstrate the approach to the case study of Malawi, a typical low-income, agrarian country susceptible to climatic shocks (WB, 2016, 2018), but that also places a strong emphasis on agriculture-led development in its national development plans (Chitiga et al. 2020). In this context, Malawi serves as a good example to analyze the role of climate change uncertainty in its prevailing development strategy.

As a modeling basis, we use a dynamic economywide computable general equilibrium (CGE) model linked to survey-based microsimulation modules that track changes in household poverty and undernourishment. We utilize this model to explore Malawi’s two long-term development strategies and their weather/climate uncertainty profiles. The first strategy reflects intensive growth within the country’s agrifood system (AFS) sectors, while its antagonist focuses on the growth of sectors outside the AFS. To represent the weather/climate-associated uncertainty of the two development scenarios, we introduce the model crop yield uncertainty block. In doing so, we use results from a probabilistic global climate model combined with historical weather variability and construct a set of crop productivity scenarios that is supplied to the CGE model. After running simulations, we conduct post-simulation estimations, construct empirical CDFs of the key outcome variables, and use SD analysis to compare the two development strategies.

Our findings indicate that despite being more vulnerable to weather/climate uncertainty and having lower and more uncertain GDP levels, the AFS-led growth strategy outperforms its non-AFS antagonist in terms of poverty reduction, both in the short term (the 2020s) and across almost all possible weather/climate scenarios in the long term (the 2040s). Additionally, the AFS strategy consistently dominates in terms of improved undernourishment levels across all potential climate/weather scenarios, suggesting that agriculture-led development remains the optimal strategy for Malawi despite increasing weather/climate risks.

The approach demonstrated in this paper has broader implications for policy planning. Irrespective of the source of uncertainty (i.e., climate, demography, world markets, etc.), many projections involve uncertainty that is often overlooked in policymaking. The incorporation of decision theory elements demonstrated in our case study thus expands the set of tools available for addressing risk and uncertainty in modern policy planning (for other methods, see e.g., Ziesmer et al. 2023; Mukashov 2023).

The remainder of this paper is organized as follows: Section 2 presents a contextual overview of the Malawian economy and its vulnerability to climate shocks. Section 3 describes the modeling framework. Section 4 discusses the results, and Section 5 concludes.

## Malawi’s economy and climate

**Fig. 1:**
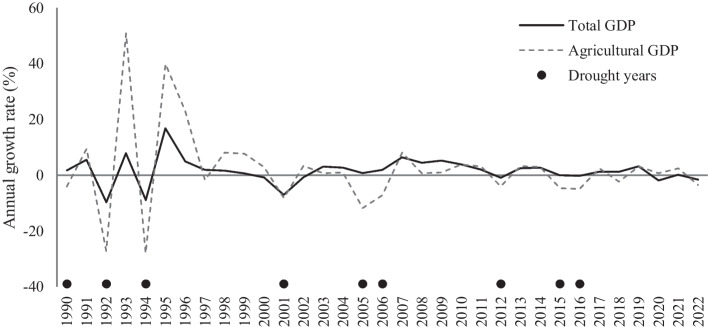
Annual GDP Growth Rates, 1991–2022. Source: Authors’ calculations using World Bank (2023)

Malawi’s economy is undergoing slow growth and structural transformation processes, with the nonagricultural economy slowly increasing its importance. The agricultural GDP and employment shares, as well as the share of the rural population, slightly declined over the last 30 years, with the primary recipient of deagrarinization and rural exodus being the urban service sector (Table 1). At the same time, the change is slow, and, similar to other less-developed African countries, Malawi is experiencing problems in generating stable growth and structural transformation. In particular, the country is characterized by relatively volatile GDP growth (Fig. 1) that is attributed to macroeconomic instability and adverse climatic shocks (WB 2018).

**Table 1:** Structure of Malawi’s Economy, 1991–2019

	1991	2004	2019
Population (million)	9.8	12.4	18.9
Urban share (%)	11.9	15.0	17.2
Poverty rate (%)	-	68.9	70.1
Undernourishment rate (%)	-	24.0	17.6
GDP per capita ($)	309.1	382.4	549.0
Total GDP (%)	100	100	100
Agriculture	34.8	30.8	24.5
Industry	30.3	19.7	19.7
Services	34.9	49.6	55.8
Total employment (%)	100	100	100
Agriculture	76.1	73.4	62.4
Industry	7.4	6.9	7.8
Services	16.5	19.7	29.8

Source: Authors’ calculations using IFPRI (2023) and World Bank (2023)

Poverty and undernourishment rates are the shares of the population whose adult-equivalent daily consumption spending is below US$2.15 (adjusted for purchasing power parity) or below the minimum calorie requirement defined by the FAO (2023)

As a result, Malawi remains one of the poorest and least developed countries in the world (WB 2018), with very low per capita GDP and consumption levels as well as high poverty and undernourishment (Table 1). Despite some signs of structural transformation, the country remains mostly rural. Primary agriculture, while constituting about a quarter of GDP, employs 62.4% of the labor force (Table 1), with most of the country’s population being smallholder subsistence farmers (WB 2018).

Despite past and existing difficulties, the Malawian government launched an ambitious “Malawi 2063” development strategy that declares the achievement of lower-middle-income status by 2030 and upper-middle-income status by 2063 as its goals (WB 2021). The formulated strategy focuses on a twin shift from subsistence to commercial agriculture and from a predominantly rural to an urbanized service and industry-driven economy (WB 2021).

At the same time, in the short and medium terms, agriculture holds a central position in national development plans, reflecting its pivotal role in addressing immediate challenges such as poverty and hunger. As a signatory to the Comprehensive Africa Agriculture Development Programme and the 2014 Malabo Declaration, Malawi has committed to achieving a 6% annual agricultural growth rate and allocating 10% of its budget to agricultural expenditure, with overarching goals of halving poverty and ending hunger. The Government of Malawi explicitly acknowledges the critical dependency of the country’s development on the agricultural sector, leading to the formulation of a national agricultural development plan that undergoes regular updates and refinements (see Chitiga et al. 2020 for a detailed overview).

In this context, our research aims to illuminate the potential vulnerability of Malawi's current prioritization of agriculture-led development—climatic uncertainty. Historically, mostly rain-fed Malawian agriculture was particularly prone to climatic shocks and demonstrated very high volatility (Fig. 1). Since 2000, the country experienced four major droughts (2005, 2012, 2015, and 2016) which had severe impacts on the entire economy. For instance, according to the World Bank, the 2015/2016 El Nino-induced droughts led to a reduction in total GDP growth of around 2.2%, making Malawi one of the worst-impacted countries in the region (WB, 2016). At the same time, as a result of lower production, the country experienced a dramatic rise in the price of staple crops like maize, which had a devastating impact on both the rural poor and urban poor (WB 2016, 2018). In this context, incorporating climate uncertainty into the discussion of Malawi’s optimal development strategy thus has high a policy priority.

## Integrated analytical framework

### Estimating direct climate impacts on agriculture

Thomas et al. (2022a, b) model the weather/climate-driven crop productivity uncertainty in 10 countries of Southern Africa. The authors use a combination of a large ensemble of climate models,^2^ historical data on an inter-annual variation in temperature and precipitation, and employ crop modeling (and emulation) methods in combination with Gaussian quadrature sampling and provide a yearly distributional estimate of the climate-driven productivity uncertainty for key crops in the region (maize, drybeans, groundnuts, soybeans, and sorghum). Then, for each crop, the authors compare the dynamics of yield distributions in the short term (2020s) and compare them with respective yield distributions in the long term (2040s and 2060s). The authors find that for many crops and countries, both medians and left tails (5th percentiles) decrease, meaning that in addition to average yields decline, one can expect increased frequency of rare low-yield events such as 1 in 20 years droughts.

**Fig. 2:**
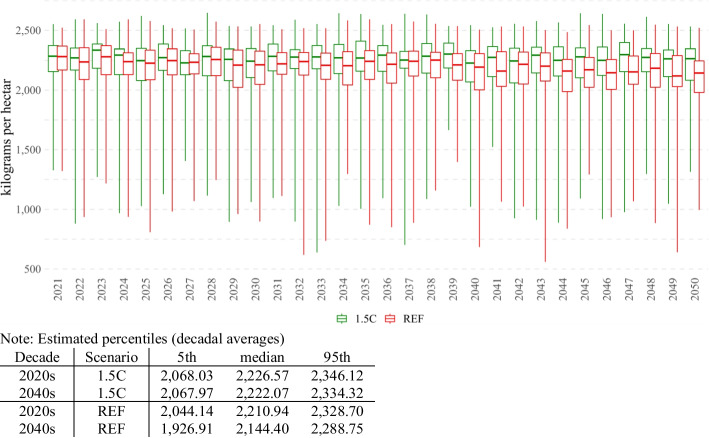
Maize yield uncertainty in Malawi. (uncertainty boxplots based on 455 weathers for each year and climate). Estimated percentiles (decadal averages). Source: Authors’ calculations based on Thomas et al. (2022b)

We use these Thomas et al. (2022a, b) estimates as a starting point for our climate-associated yield variation scenarios for Malawi. Figure 2 represents the example of maize yield distribution estimates (in kg per hectare, similar estimates are available for drybeans, groundnuts, soybeans, and sorghum). For the sake of parsimony, in this paper, we focus on two polar cases of global climate change scenarios—an 'optimistic' scenario (1.5C) that assumes global warming is kept to 1.5 °C above pre-industrial levels, and a 'pessimistic' scenario (REF) that assumes no explicit climate mitigation policies are implemented anywhere in the world. Figure 2 then represents the estimated dynamics of maize yield distribution for every climate and year (each year and climate have a sample of possible 455 weathers, each of which has its own probability).

We cannot use these yield estimations per se and have to conduct several preprocessing steps to properly define scenarios for our Malawian CGE model.

First, unlike crop models, our CGE model assumes nonconstant (increasing) Total Factor Productivity (TFP). In this context, we decide to adopt yield estimates in relative terms. To do so, we create a hypothetical No GCC (No Global Climate Change) scenario that corresponds to the weighted average median yields of the initial year for which weather/climate yields are available (2021). No GCC scenario then serves us as a reference (as if there is no weather/climate-driven variation), and we normalize to it the kg per hectare estimates. Second, given data and modeling restrictions, crop models provide estimates for six key crops only (maize, drybeans, groundnuts, soybeans, and sorghum). Therefore, to properly reflect weather/climate-driven productivity variation of the entire crop sector of Malawi, we impute the climate-driven yield variability for remaining crops. In doing so, we use FAO estimations on historical yields and apply regression methods (a detailed explanation is given in Appendix A).

**Fig. 3:**
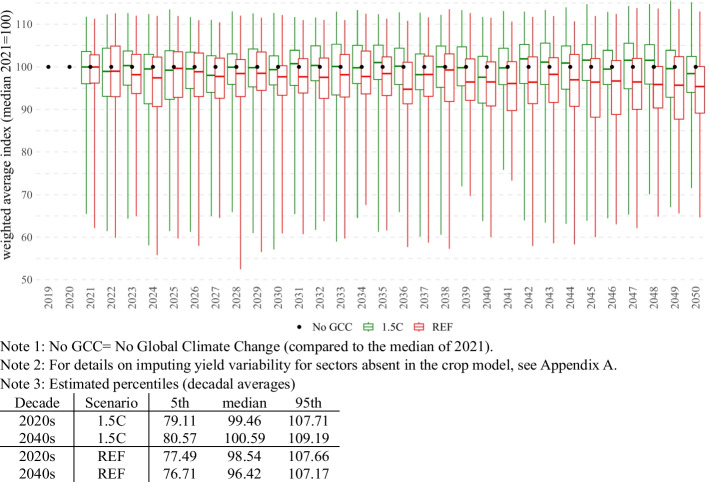
Yield variation of the entire crop sector in Malawi, (uncertainty boxplots based on 455 weathers for each year and climate). 1: No GCC = No Global Climate Change (compared to the median of 2021) 2: For details on imputing yield variability for sectors absent in the crop model, see Appendix A 3: Estimated percentiles (decadal averages). Source: Authors’ calculations

Then, even before referring to the CGE model, the obtained information on the yield uncertainty allows us to conduct the first-level analysis of the severity of climate change in Malawi. In particular, assuming constant value-added shares, it is possible to construct a yearly weather/climate-associated yield uncertainty of the entire crop sector (Fig. 3). In this context, it is worth noting that under less severe climate scenario 1.5C, the performance of the entire crop sector in Malawi is expected to be moderately affected by weather/climate-driven factors (as evidenced by almost the same percentiles in the 2020s and 2040s). However, the impact of the climate is more pronounced under a more severe climate REF, whose both median and 5th percentiles in the 2040s are considerably lower than in the 2020s. Given that the 95th percentile almost does not change, we can thus conclude that weather/climate-associated productivity uncertainty in Malawi increases over time, and this increase can be primarily attributed to the increased probability of low-yield events such as droughts.

### Modeling economywide climate impacts

As a modeling basis, we use the standard recursive-dynamic CGE model of the International Food Policy Research Institute (IFPRI) and its satellite poverty and undernourishment microsimulation modules. As its corresponding data inputs, we use the 2019 Social Accounting Matrix^3^ (SAM) (IFPRI, 2023) and the 2019/2020 household survey (National Statistical Office of Malawi, National Statistical Office of Malawi 2021). Given the space constraints of this paper, in what follows, we briefly outline key aspects of the model and focus on the specific adjustments we make to simulate Malawi’s development under weather/climate uncertainty.

#### Core CGE

*.* The IFPRI CGE model allows for a macroeconomically consistent representation of economic linkages between activities, households, and the rest of the world, and its completely endogenized prices of products and factors (land, labor, capital) reflect resource competition on all levels. Given these advantages, the model is a workhorse tool that is routinely used to simulate country-level economy-wide adjustments in response to various scenarios, including climate change studies (e.g. Arndt and Thurlow 2014, Arndt et al. 2014 and Siddig et al. 2020). The detailed statements of the CGE model equations can be found in Löfgren et al. (2002), and Diao and Thurlow (2012), and the description of satellite poverty and undernourishment microsimulation modules can be found in Pauw and Thurlow (2011).

#### Base year SAM

The constructed SAM utilizes the latest available information and reflects the detailed snapshot of the flows across the Malawian economy for the year 2019 (which accordingly, becomes the base/start year of our simulations). In particular, our SAM consists of 203 accounts, of which 82 are activities, 83 are commodities, 13 are primary factors (labor, capital, and land), and 15 are households.^4^

#### (Implicit) BAU scenario

We select 2050 as our simulation horizon, and in the context of mediocre and volatile past performance (see Fig. 1), as well as concerns expressed in the Systematic Country Diagnostic report by the World Bank regarding the country’s growth prospects (WB, 2018), we assume that under the (implicit) BAU scenario, the country will grow at approximately 1% per annum (p.a.) per capita, with the TFP growth rates across all sectors being 1% p.a.

#### Development scenarios

Then, instead of BAU TFP growth of 1% p.a., our development scenarios assume weighted average (economywide) TFP growth of 2% p.a. Consequently, the difference between the two alternative scenarios is the sectoral distribution of the additional TFP growth. In particular, under the AFS development, we assume that the additional 1% of economywide TFP growth is achieved via the sectors associated with agriculture (uniform distribution across primary agriculture, agro-processing, and food services (e.g. hotels and restaurants)). Alternatively, under the non-AFS-led development scenario, we assume that the TFP boost is achieved via the sectors associated with (other) industrial and service sectors (except public administration). Then, because of the different means of achieving similar economy-wide TFP growth, the two scenarios can be compared across the dimensions of their varying outcomes.^5^

#### Weather/climate uncertainty

The weather/climate uncertainty block consists of 455 yearly weather scenarios for each of the two climates. Importantly, we assume that in the long term, the reallocation of factors in the economy follows the long-term fundamental factors as if there is no climate/weather uncertainty. Then, weather/climate uncertainty is modeled as a possible variation around the long-term development trajectory with constant weather/climate. Importantly, we assume that land in the long term adjusts according to the long-term fundamentals, but land becomes immobile across agricultural activities *within* years. In other words, we assume that the land is (re)allocated in line with long-term rents and short-term weather/climate fluctuations do not affect this fundamental adjustment process.

**Fig. 4:**
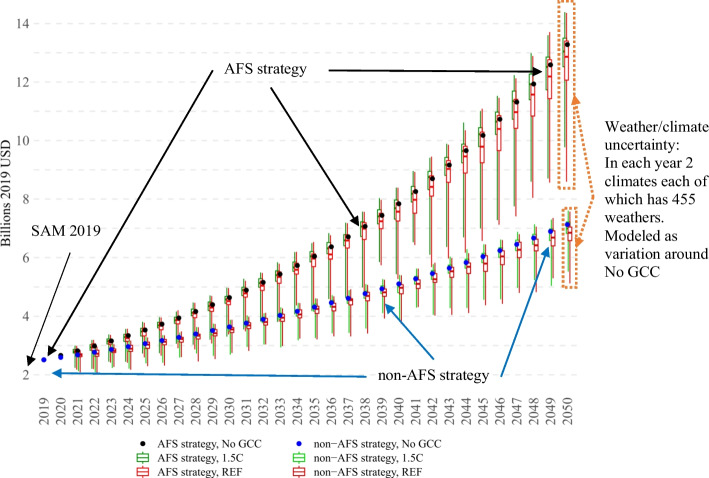
Design of model simulations. Agricultural GDP, uncertainty boxplots based on 455 weathers for each year, and climate. Source: Authors’ elaborations

Figure 4 represents a schematical representation of our simulations’ design. In particular, it represents the *yearly* distributions of agricultural GDP of two development strategies under weather/climate uncertainty. Most importantly, it should be noted that we do not use the standard counterfactual CGE analysis approach (where outcomes of the baseline scenario are used as a reference). Instead, because we are focused on comparing *uncertainties*, we use absolute levels. In this context, the presented uncertainty dynamics of agricultural GDP can help to better understand the complexity of the problem of comparing two strategies. In particular, because AFS promotes agriculture, and non-AFS promotes other sectors, in terms of agricultural GDP, the two *uncertainties* eventually diverge, and the intersection between them becomes impossible (in the long term). This allows the conclusion that AFS unambiguously outperforms non-AFS in terms of agricultural GDP results. However, other more important outcome variables such as total GDP per capita, poverty, or undernourishment, might not allow for unambiguous ranking, which facilitates the necessity to use CDF and SD analysis methods.

#### Post-simulation imputation of uncompleted simulations

Finally, it should be noted that in some instances our CGE model formulated in General Algebraic Modeling System (GAMS) cannot find a (feasible) numeric solution. Most of the time the model’s inability to solve is associated with the magnitude of negative yield shocks of particular crops (e.g., in some years, extreme scenarios assume more than 90% yield reduction). To accommodate these shocks, our specified CGE model has to look for solutions in the unfeasible domain.^6^ Therefore, to avoid truncation of the unsolved cases (depending on the year and climate, can reach ca. 1% of weather scenarios), we decided to impute missing/unsolvable cases. In doing so, we use the following approach: 1. For each year and climate scenario, we select the lowest 25 percentile of complete cases 2. We (linearly) regress outcome variables on the weighted average crop weather/climate index of complete cases (the explanatory power of this simple CGE emulator, depending on the year, scenario, and the outcome variable, varies from R sq. = 0.68 to R. sq. 0.95). 3. We project outcomes of the unsolved simulations using their weighted average crop climate/weather index (for the unsolved year and climate) and the estimated relationship. We suppose that this ad-hoc approach is sufficient to conduct the *comparison* of the distributions of our outcome variables. At the same time, it can be noted that this imputation approach might be inappropriate if the behavior of the economic system under the extreme case scenarios itself becomes a goal of a study (in this case, one would require an entirely different modeling approach which is beyond the focus of this study).

## Simulation results

**Fig. 5:**
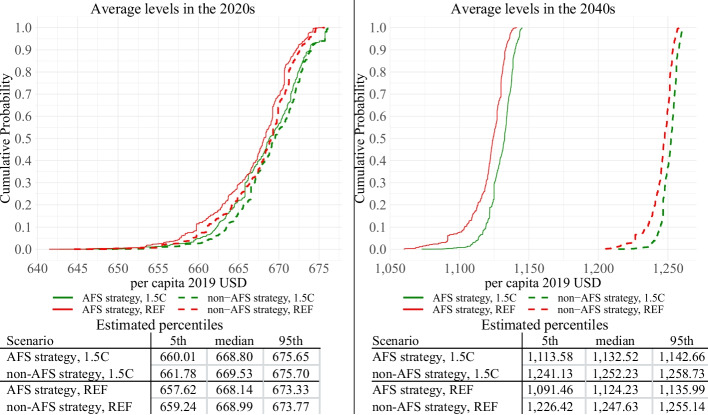
Simulation results—GDP per capita. Empirical CDFs. Source: Authors’ calculations

**Fig. 6:**
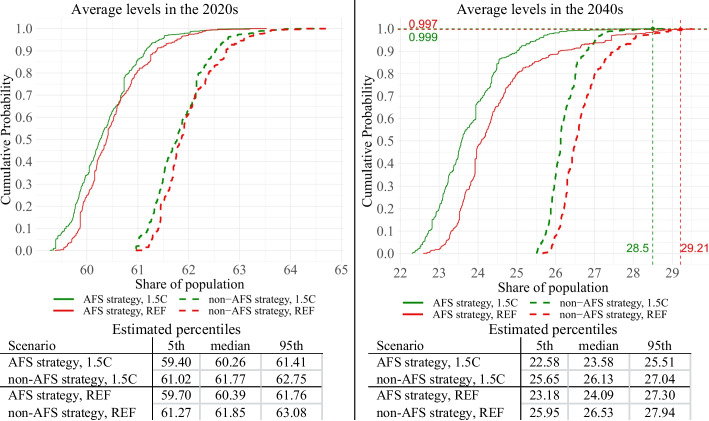
Simulation results – national poverty rate. Empirical CDFs. Source: Authors’ calculations

**Fig. 7:**
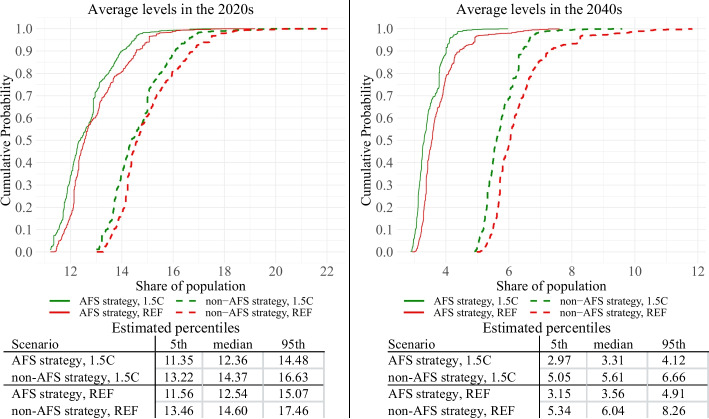
Simulation results – national undernourishment rate. Empirical CDFs. Source: Authors’ calculations

Given our focus on Malawi’s national developmental goals, we analyze the results associated with three key outcome variables – real per capita GDP, poverty, and undernourishment rates. These results are depicted in Figs. 5, 6 and 7. These figures represent estimated empirical CDFs of average decadal levels (2020s and 2040s) of each development strategy and climate; below each figure, we also present calculated 5th, 50th, and 95th percentiles.

It should be also noted that we use the term ‘stochastic dominance’ to refer to first-order stochastic dominance. By definition, first-order stochastic dominance of X over Y means that X must have at least as high a probability of reaching a particular outcome as Y for any given uncertainty realization (for details, see Levy 1992). In the context of our study, ‘stochastic dominance’ implies that strategy X has a *better* outcome than strategy Y, with ‘better’, depending on the context, meaning higher outcomes (e.g., per capita GDP) or lower outcomes (e.g., poverty levels).

As can be seen in Fig. 5, the non-AFS strategy is associated with higher GDP per capita levels. Under the non-AFS development, both climates (dashed) stochastically dominate their AFS counterparts (solid). In the 2020s, the differences are marginal (depending on the weather and climate, in the 2020s non-AFS outperforms AFS by 0%-0.51%); however, by the 2040s, the difference widens and reaches 10.01%-13.70% (non-AFS’ outperformance depends on the weather and climate). The stochastic dominance of non-AFS can be attributed to the fact that its sectors have more substantial downstream economy-wide linkages, and their increased productivity has higher indirect benefits, especially in the long term. For instance, increased productivity of important industrial intermediates as well as trade and transport sectors means that all sectors in the economy benefit from increased production and lower prices. Similarly, higher productivity and lower prices of non-AFS investment sectors (e.g., construction) particularly benefit capital accumulation, resulting in higher GDP levels in the long term.

At the same time, it should be noted that AFS uncertainty is higher than that of non-AFS. Under the AFS strategy, by the 2040s, the GDP difference between the best and worst weather conditions (95th versus 5th percentiles in Fig. 5) reaches 2.61% and 4.08% for 1.5C and REF climates respectively; this variation is almost two times higher than 1.42% and 2.34% for 1.5C and REF climates under non-AFS strategy. This is because the two strategies have different impacts on the economic weight of the agricultural sector, which is the driving force of the weather/climate uncertainty in our model. Specifically, the non-AFS strategy over the years reduces the share of the crops from 20.43% of GDP in 2019 to 12.51% in 2050, while AFS increases the share to 26.13%. As a result, the two strategies lead to two different economies characterized by different exposures to weather/climate uncertainty, with the non-AFS economy being less exposed. In this context, it’s also worth noting that although the variation between the two climates within the same development scenario is marginal (1.5C versus REF), AFS and non-AFS strategies have important differences. In particular, although in both AFS and non-AFS, the 1.5C scenario is always better than REF, AFS is characterized by a higher climate sensitivity. Depending on the weather scenario, in the 2020s, the difference between 1.5C and REF under AFS development varies from 0% to -0.5% (compared to 0%-0.4% for non-AFS), and by the 2040s, it increases to -0.3% to -3.1% (compared to -0.1% to -1.8% for non-AFS).

Poverty results (Fig. 6) do not repeat GDP per capita outcomes, and in general, AFS tends to outperform non-AFS. For instance, under median weather conditions (cumulative probability = 50), AFS outperforms non-AFS, especially in the long term. Similar to other studies (e.g. Diao et al. 2010, Valdes and Foster, 2010; Klasen and Reimers 2017) this result can be primarily attributed to deeper pro-poor linkages of agriculture. In the context of our CGE model, the following drivers of this result can be highlighted: 1. Increased TFP of agricultural sectors directly raises the incomes of rural farmers, who are the poorest in Malawi 2. Increased production leads to cheaper food prices which particularly benefit both urban and rural poor 3. Increased TFP of AFS sectors means that AFS sectors require less labor, enabling the agricultural labor force to shift to urban sectors where their labor is the most needed (in other words, increased TFP of AFS has a more pronounced effect on the structural transformation).

However, analysis of the whole weather/climate uncertainty range complicates the picture. As the share of crop sectors is growing under the AFS strategy, the economy is becoming more exposed to weather/climate uncertainty, and poverty levels are becoming more uncertain. By the 2040s, under the most favorable weather/climate conditions, the poverty rate of AFS can be as low as 22.30%, and under the worst conditions, the poverty rate can be as high as 29.58%. This range is wider than non-AFS, which ranges from 25.49% to 29.51%. In the 2020s, AFS has robustly lower poverty rates than non-AFS, but as the AFS economy is becoming more exposed to weather/climate shocks, and as the magnitude of the shocks is becoming more severe (see Fig. 3(, by the 2040s, AFS is no longer robustly superior. Under very extreme worst-case weather, poverty under the non-AFS development can be lower than that of AFS. Specifically, under the 1.5C scenario, AFS intersects non-AFS at a 0.999 cumulative probability, and under the REF scenario, the intersection is at a 0.997 cumulative probability. Assuming both 1.5C and REF climate scenarios are equally possible, we can calculate an average of 0.9985 cumulative probability and conclude that for 99.85% of possible weather/climate scenarios, AFS will have better poverty results than non-AFS. However, under 0.15% of scenarios (with extremely low crop yields), the non-AFS economy will have lower poverty than the AFS economy,

It should be also noted that the distance between the two strategies is not constant. In particular, in the 2040s, under the worst 5% weathers (cumulative probability = 0.95), the difference between AFS and non-AFS is 1.53% for the 1.5C climate and only 0.25% for the REF climate; under median weather (cumulative probability = 0.5), the difference between AFS and non-AFS reaches 2.55% for 1.5C and 2.04% for REF climates; under the most favorable weather (cumulative probability = 0), difference reaches 3.21% and 2.91% for 1.5C and REF respectively. In other words, the rate of AFS outperformance depends on climate/weather conditions, and the further the weather/climate will be from negative extremes, the better the AFS’s outperformance in terms of poverty reduction.

In terms of undernourishment level results (Fig. 7), AFS unequivocally outperforms non-AFS (both in the short and long term). This is mostly because the productivity boost of AFS sectors leads to increased domestic production and a consequent decrease in food prices, which improves food accessibility (as a result, the rise in calorie intake among undernourished households is much higher). The decline in undernourishment rates under the AFS strategy is so substantial that the escalating severity of climate/weather scenarios is not sufficient to notably affect its undernourishment levels. Thus, both in the short and long term, AFS is robustly superior (stochastically dominant) to non-AFS.

At the same time, it is noteworthy that the distance between respective CDFs is relatively constant for both decades and scenarios except for REF in the 2040s. Deterioration of non-AFS undernourishment levels under more severe weathers of the REF climate in the 2040s becomes particularly pronounced, and under 5% of the most extreme weathers, non-AFS can have undernourished levels two times higher than its AFS counterpart (cumulative distribution > 0.95 in the 2040s). In summary, it can be concluded that AFS is guaranteed to yield lower undernourishment levels, and under very negative weather/climate scenarios, the outperformance of AFS can be very substantial.

## Conclusion

Our study uses an innovative approach to comparing two potential development strategies under the uncertain weather/climate projections for Malawi, a country heavily reliant on the agricultural sector. Specifically, we use elements of SD theory to examine the AFS and non-AFS growth strategies’ economic uncertainty driven by weather/climate-associated productivity variation.

We demonstrate that despite higher economic uncertainty, AFS development leads to lower poverty and undernourishment levels (exhibiting stochastic dominance across almost all weather and climate scenarios). It is crucial to note that we model variation of weather/climate-related productivity of crop sectors only, and we do not consider potentially buffering climate adaptation measures within agriculture itself. This means that overall, we might overestimate the relative uncertainty of AFS development. However, even with that, AFS outperforms non-AFS in terms of both poverty and undernourishment. In the context of the multifaceted vision of the 'Malawi 2063' national development plan, our findings thus underscore the clear benefits of prioritizing an agriculture-led development path for Malawi.

In light of these findings, it becomes essential to pursue further deeper-level research and consider trade-off aspects of climate adaptation options within agriculture itself. Several micro-level studies have already begun to investigate specific aspects of various climate adaptation options in Malawi's agricultural sector. For example, Amadu et al. (2020a) developed a typology of farm-level climate-smart practices in southern Malawi, and Amadu et al. (2020b) analyzed the effects of climate-smart agricultural aid investment on maize yields. Sitko et al. (2021) used household-level data and analyzed climate-adaptive agricultural practices in the context of households’ risk perception leverage through receiving food aid. At the same time, such studies, while offering valuable empirical evidence on certain climate adaptation methods, cannot fully reflect a complex picture of policy return and risk trade-offs. A relevant example is the study by Warnatzsch and Reay (2020), who used a crop model to highlight how the uncertainty about future precipitation is associated with maladaptation risk for maize cultivars in Malawi. The authors stressed the need for more reliable precipitation projections for proper decision-making. However, given the inherent uncertainty of weather and climate scenarios themselves, the emergence of 'robust precipitation projections' is doubtful. In this context, our suggested methodology of comparing uncertain competing options via the means of CDF and SD analysis can be extended and used in future deeper-level policy modeling studies.

## Data Availability

The Social Accounting Matrix used in this study is available in the Harvard Dataverse repository, 10.7910/DVN/C9WA0I. The household survey used in this study is available in the World Bank Microdata Libraryrepository, 10.48529/yqn3-zv74.

## Appendix A. Imputing climate-driven yield variability for missing crops

Tables 2 and 3

Crop-yield models used by Thomas et al. (2022b) provide us with weather/climate-associated crop yield variation scenarios for five key crops: maize, drybeans, groundnuts, soybeans, and sorghum. Together, these crops constitute 52.02% of the agricultural value added in 2019. Accordingly, we need to impute yield variation scenarios for the remaining 47.98% of crops that are not captured by the crop yield models (Table 2).

**Table 2:** Crops with available and imputed scenarios

Category	Agricultural subsector	CGE label	Value-added %
Covered by crop-yield models	Maize	amaiz	33.67
	Drybeans	apuls	10.12
	Groundnuts	agnut	5.50
	Soybeans	aoils	1.81
	Sorghum	asorg	0.92
	Total		52.02
Not covered by crop-yield models -Imputed	Rice	arice	4.43
	Cassava	acass	5.11
	Irish potatoes	aipot	2.03
	Sweet potatoes	aspot	3.87
	Leafy vegetables	aleaf	13.85
	Other vegetables	avege	5.38
	Sugarcane	asugr	2.49
	Tobacco	atoba	1.98
	Cotton and fibers	acott	0.25
	Nuts	anuts	0.40
	Bananas	abana	1.97
	Plantains	aplan	0.65
	Other fruits	afrui	4.13
	Leaf tea	ateal	1.03
	Coffee	acoff	0.18
	Other crops	aocrp	0.24
	Total		47.98

Source: Authors’ calculations

To impute the climate-driven yield variability for the remaining crops, we rely on FAO estimations of historical yields in Malawi (FAO 2023) and adhere to the following conventions:

1. The historical range is 2003 to 2020, as data for many crops before 2003 is unavailable.
2. Our SAM has aggregated accounts for certain groups of crops (e.g., vegetables). To approximate these crops, we select the FAO crops with the most consistent and reliable data. For example, as a proxy for yield variation of the aggregate vegetable sector, we choose FAO data on green peas (other more important vegetables, like tomatoes, have data consistency problems).
3. Our focus is solely on weather/climate-associated yield variation. Hence, we filter out linear trends from FAO yield estimates and assume that historical yield variation around that linear trend was driven by weather/climate factors.
4. Similar to crops with available scenarios, we normalize the yields and employ relative indices (with 100 indicating no impact from weather/climate factors and the yield being on the historical TFP growth trend).
5. As our primary objective is to specify climate-driven yield variability (without drawing any foundational inferences), we concentrate exclusively on prediction accuracy and employ the software Eureqa, which is tailored for evolutionary search across various analytic equation forms to get the optimal fit (for a concise overview of the software, refer to Dubčáková 2010, and Schmidt and Lipson 2009). In weighing accuracy against overfitting, we made an ad-hoc decision and limited models to full quadratic polynomial regressions, where endogenous variables represent missing crops and covariates include maize, drybeans, groundnuts, soybeans, and sorghum. As a result, we selected the following regressions:

**Table 3:** Imputation formulas of missing crops

CGE label	FAO	Formula/note	R^2
crice	Rice	crice = 3.67 + 0.59*coils + 0.27*csorg + 0.12*cmaiz	0.85
ccass	Cassava, fresh	ccass = 98.53 + 0.0026*cgnut^2—0.25*coils	0.62
cipot	Potatoes	cipot = 51.95 + 0.49*coils	0.64
ctoba	Unmanufactured tobacco	ctoba = 39.51 + 0.61*cgnut	0.75
cplan	Plantains and cooking bananas	cplan = 80.21 + 1.40*csorg + 1.07e-6*csorg^2*coils^2—0.009*csorg*coils—0.0001*coils*csorg^2	0.66
cnuts	Other nuts n.e.c	cnuts = 114.70 + 0.11*csorg + 0.10*cmaiz + 0.0028*cpuls^2—0.48*cpuls—0.0012*csorg*puls—0.0003*cmaiz^2	0.63
cleaf	Cabbages	cleaf = 215.46 + 0.287*cpuls + 0.19*csorg + 0.03*cmaiz*cgnut—2.87*cgnut—0.006*cmaiz*cpuls—0.01*cmaiz^2	0.55
cvege	Peas, green	cvege = 85.12 + 0.66*coils + 0.04*csorg*cgnut + 0.003*cpuls*cgnut—2.29*csorg—0.009*cgnut^2—0.0002*csorg*cgnut^2	0.81
ccott	Seed cotton, unginned	ccott = 227.25 + 0.0008*csorg*coils^2—0.05*coils^2—0.0004*coils*csorg^2	0.74
cfrui	Other fruits, n.e.c	cfrui = 99.86 + 0.02*coils + 0.0002*cgnut^2—0.001*cmaiz—0.01*cgnut—0.0002*cgnut*coils	0.53
cocrp	Other stimulant, spice, and aromatic crops, n.e.c	cocrp = 81.10 + 0.05*csorg + 0.0003*cpuls + 0.004*cpuls*coils + 4.13e-5*csorg*cgnut*coils—2.94e-5*cpuls*csorg^2—4.06e-5*cgnut*coils^2	0.76
csugr	Sugar cane	FAO data problems—use cocrp formula	-
cteal	Tea leaves	FAO data problems—use cocrp formula	-
ccoff	Coffee, green	FAO data problems—use cocrp formula	-
cbana	Bananas	FAO data problems—use cplan formula	-
cspot	Sweet potatoes	FAO data problems—use cipot formula	-

Source: Authors’ calculations

## Footnotes

1 Examples include Bansal et al. (2013), who analyzed the consistency of rankings between genetically modified and non-genetically modified food based on experimental evidence from India, and Akpalu and Ametefee (2014), who investigated self-serving bias among illegal fishers in Ghana.

2 Coupled Model Intercomparison Project (CIMP) 5. See Thomas et al. (2022a, 2022b) for details.

3 A detailed explanation of the SAM data concept can be found in Pyatt and Round (1985) and Breisinger et al. (2009).

4 The constructed SAM is available from the authors upon request.

5 The strong implicit assumption is that in Malawi the technological development is homogenous across all sectors, and that sectors of the same size for the same budget will get the same TFP increase.

6 E.g., turn the value-added activity price of the affected crop sector negative.
